# The changing face of Irish head and neck cancer epidemiology: 20 years of data

**DOI:** 10.1007/s00405-021-07118-4

**Published:** 2021-10-13

**Authors:** Gerard P. Sexton, Paul Walsh, Frank Moriarty, James Paul O’Neill

**Affiliations:** 1 Department of Otolaryngology-Head and Neck Surgery, Beaumont Hospital, Beaumont Road, Dublin 9, Ireland; 2 Royal College of Surgeons in Ireland, Dublin, Ireland; 3 National Cancer Registry Ireland, Cork Airport Business Park, Cork, Ireland; 4 School of Pharmacy and Biomolecular Sciences, Royal College of Surgeons in Ireland, Dublin 2, Ireland

**Keywords:** Head and neck surgery, Epidemiology, Laryngeal cancer, Oral cavity cancer, Nasal cavity cancer, Nasopharyngeal cancer, Oropharyngeal cancer, Hypopharyngeal cancer, Cancer survival, Elderly head and neck cancer

## Abstract

**Background:**

Head and neck cancer (HNC) is associated with significant morbidity and mortality, especially when high stage disease is present. The epidemiology and prognosis of HNC has changed considerably over the last 20 years.

**Aims:**

This study aimed to examine the epidemiological trends in HNC patients over a prolonged period in Ireland.

**Methods:**

We conducted a retrospective cohort study using 20 years of cancer registry data provided by the National Cancer Registry of Ireland. Baseline characteristics and survival statistics were thereby generated.

**Results:**

10,148 patients were identified. There is a growing population of young (< 50 years) and very old (> 85 years) HNC patients; 48.15% of the population was elderly (> 65 years). Oral cavity (29.8%) and laryngeal cancer (28.1%) remain the most prevalent subsites, though oral cavity cancer prevalence declined from 35.9% in 1994 to 27.5% in 2014. Oropharyngeal cancer prevalence increased from 13.6 to 22.2% over the same period. Overall 5-year survival has improved significantly to 56.8% in 2010 but there remains a disparity between the elderly and adult cohorts (42.0% vs 60.7%). 5-year survival for hypopharyngeal and oropharyngeal cancers has improved from 11.8% and 33.3% to 22.2% and 44.8%, respectively, while laryngeal and oral cavity cancer survival remains approximately stable at 58.7% and 61.5%, respectively.

**Conclusion:**

HNC survival in Ireland has improved in line with increasing recognition of the value of multidisciplinary assessment, subspecialisation in cancer care, and targeted therapies based on tumour subsites. Survival in the elderly cohort remains poor despite increasing recognition of the challenges such cases pose.

## Introduction

Head and neck cancer (HNC) is a heterogeneous group of malignancies comprised of cancers of the oral cavity, oropharynx, nasopharynx, hypopharynx, larynx, salivary glands, nasal cavity, and paranasal sinuses [1]. HNC accounts for approximately 6% of new cancer diagnoses worldwide [2]. Approximately a quarter of HNC occurs in elderly patients [3] with age and comorbidity often highlighted as prognostic predictors due to their effect on therapeutic tolerance [4]. There is also a significant association between HNC and socioeconomic deprivation [5]. The primary histopathological diagnosis is Squamous Cell Carcinoma (SCC), comprising well over 90% of cases [6], with a heavy preponderance of this patient group towards prolonged exposure to tobacco, alcohol, or both [7]. Human Papilloma Virus (HPV) infection has been increasingly recognised over the last 30 years as a major risk factor for the development of HNC, with oropharyngeal malignancy displaying such a strong association that HPV status now forms part of the staging process for such patients [1, 8–11]. There is a further association between nasopharyngeal malignancy and Epstein–Barr Virus (EBV) [12, 13]. Up to 70% of HNC presents with either locally advanced or metastatic disease in the first instance [14]. There has up to this point been no aggregated analysis of the epidemiological data of Irish HNC patients.

## Aims and objectives

This study aimed to examine the epidemiological trends in HNC patients over a prolonged period in Ireland. Specific objectives included determining the overall survival by stage, age, year of incidence, and location.

## Materials and methods

### Study design

A retrospective cohort study was conducted using STROBE standardised reporting guidelines. The study cohort was derived from a database obtained from the National Cancer Registry of Ireland (NCRI) of HNC patients diagnosed in Ireland between 1994 and 2014. The length of cancer-specific follow-up in this instance is until the end of 2015. This database was derived from both electronic healthcare records and physical charts which the NCRI analyses on a continual basis. The elderly population was predefined as anyone over 65 years of age. This age was chosen as the most often quoted definition of elderly in published literature both in HNC and in general [4].

### Inclusion/exclusion criteria

The sole inclusion criterion was patients with HNC (as defined by TNM Classification of Malignant Tumours (TNM) for Head and Neck Cancer 8e [1]) diagnosed within the period specified. The primary exclusion criterion was patients with cancers occurring outside this region. Cutaneous and thyroid malignancies were also excluded owing to the considerable differences they display in terms of treatment and prognosis.

### Statistical methods

Descriptive statistics for included participants’ baseline characteristics were generated. Year of incidence data by site and age, overall stage at presentation statistics, and 5-year survival statistics by stage, year of incidence and site were also generated using Stata version 16.1.

### Ethical considerations

Ethical approval was sought from and approved by the RCSI Research Ethics Committee. The database in question already exists, and the NCRI retains legislative authority to analyse data for research, and release data to external parties specifically for this purpose [15]. All data derived from the NCRI database are fully anonymised in line with best practice as outlined by the Data Protection Commission, and their lawful grounds for processing same is pursuant to compliance with a legal obligation [15]. Informed consent has not been explicitly sought from any of the patients involved.

## Results

### Baseline characteristics

Between January 1994 and December 2014, there were 10,148 HNC patients in the Republic of Ireland. The baseline characteristics of these patients are summarised in Table 1.

**Table 1:** Baseline characteristics of Irish head & neck cancer patients 1994–2014

Variable	Frequency	Percentage	Cumulative %
Age group			
00_04	11	0.11	0.11
05_09	12	0.12	0.23
10_14	23	0.23	0.45
15_19	33	0.33	0.78
20_24	30	0.3	1.07
25_29	46	0.45	1.53
30_34	106	1.04	2.57
35_39	161	1.59	4.16
40_44	297	2.93	7.09
45_49	649	6.4	13.48
50_54	990	9.76	23.24
55_59	1,375	13.55	36.79
60_64	1,529	15.07	51.85
65_69	1,441	14.2	66.05
70_74	1,270	12.51	78.57
75_79	997	9.82	88.39
80_84	674	6.64	95.03
85+	504	4.97	100
Year of incidence			
1994	434	4.28	4.28
1995	431	4.25	8.52
1996	417	4.11	12.63
1997	405	3.99	16.62
1998	427	4.21	20.83
1999	397	3.91	24.74
2000	414	4.08	28.82
2001	319	3.14	31.97
2002	440	4.34	36.3
2003	422	4.16	40.46
2004	452	4.45	44.92
2005	468	4.61	49.53
2006	482	4.75	54.28
2007	489	4.82	59.1
2008	504	4.97	64.06
2009	536	5.28	69.34
2010	603	5.94	75.29
2011	603	5.94	81.23
2012	601	5.92	87.15
2013	661	6.51	93.66
2014	643	6.34	100
Gender			
F	2,623	25.85	25.85
M	7,525	74.15	100
Histology			
Adenocarcinoma	631	6.22	6.22
Melanoma	61	0.6	6.82
Unspecified	825	8.13	14.95
Sarcoma	76	0.75	15.7
Squamous cell carcinoma	8,555	84.3	100
Site			
Hypopharynx	793	7.81	7.81
Larynx	2,848	28.06	35.88
Nasal cavity/paranasal sinuses	482	4.75	40.63
Nasopharynx	305	3.01	43.63
Oral cavity	3,028	29.84	73.47
Oropharynx	1,606	15.83	89.3
Other	366	3.61	92.91
Salivary gland	720	7.09	100

Elderly patients accounted for 48.1% (*n* = 4886) of cases. The incidence of HNC in Ireland remained stable for the first 10 years of the study period but has subsequently risen year on year, with 434 cases in 1994 compared to 643 cases in 2014. 74.2% (*n* = 7525) of patients were male. The predominant histopathological diagnosis was SCC in 84.3% (*n* = 8555), though there was a notable proportion of malignancies coded as “unspecified carcinoma” (*n* = 825, 8.13%). Adenocarcinoma was the next most commonly occurring diagnosis in 6.2% (*n* = 631).

There were 18 sites and 79 subsites reported in the dataset. These were analysed and grouped according to anatomical definitions laid out by the AJCC [16] into the seven specific groups detailed in Table 1. Lesions with sites that overlapped these groups or which were without a definite anatomical location were designated “other” (*n* = 366, 3.6%). Oral cavity cancer was the most common malignancy in 29.8% (*n* = 3028) followed closely by laryngeal cancer in 28.1% (*n* = 2848) and oropharyngeal in 15.8% (1606). When broken down by gender, this pattern was replicated in female patients (36.5%, 16.9%, 14.6%). In male patients, the larynx was the most commonly affected site (32.2%, *n* = 2423), followed by the oral cavity (27%, *n* = 2032).

Year of incidence data organised by disease site is presented in Table 2. The incidence of HNC rose across all disease sites, with the oral cavity, larynx, and oropharynx consistently the most prevalent. There was a decrease in the relative prevalence of oral cavity cancer from 35.9% in 1994 to 27.5% in 2014. There was also an increase in the relative prevalence of oropharyngeal cancer from 13.6% in 1994 to 22.2% in 2014.

**Table 2:**
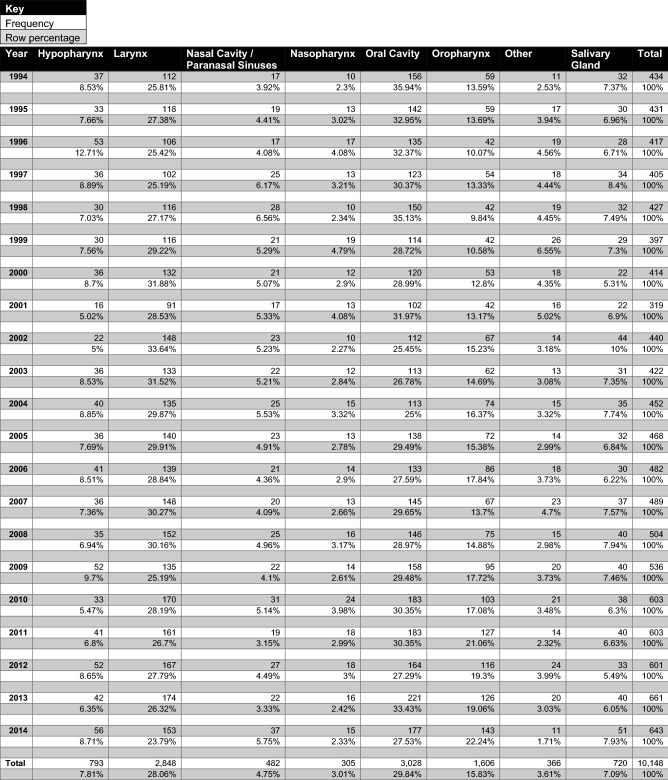
Year of incidence head and neck cancer data organised by site

Year of incidence data organised by age group is presented in Table 3. The proportion of patients over 65 years changed over the 20 years observed—this group comprised 56.4% of cases in 1994 and 46.8% in 2014. Despite this there was a steady rise in the prevalence of HNC in patients over 85 years (3.9% in 1994 to 5.3% in 2014). The prevalence of HNC in patients over 50 years and less than 65 years increased over the 20 years observed from 33.4% in 1994 to 38.1% in 2014. A similar increase was seen in patients less than 50 years (10% in 1994 vs. 15.2% in 2014).

**Table 3:**
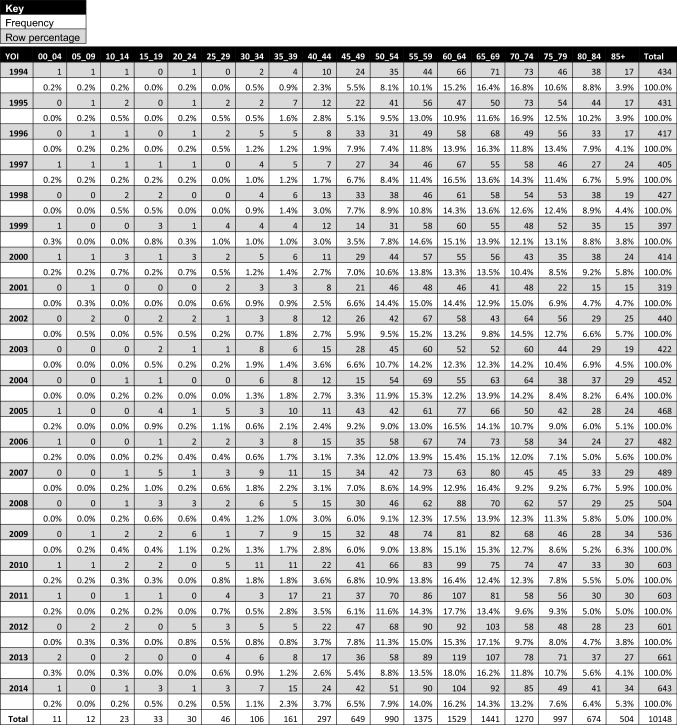
Year of incidence head and neck cancer data organised by age group

### Stages and survival

Stage at presentation statistics overall and organised by site are summarised in Table 4. Patients presenting with stage 0, stage I or stage II disease comprised 30.3% (*n* = 3075). Stage III disease was present in 10.9% (*n* = 1102), non-metastatic stage IV disease was present in 24.3% (*n* = 2469), and metastatic stage IV (stage IVC) disease was present in 3.9% (393). Oropharyngeal and hypopharyngeal cancers in particular presented with advanced disease in at least 63.3% and 44.4% of cases, respectively.

**Table 4:**
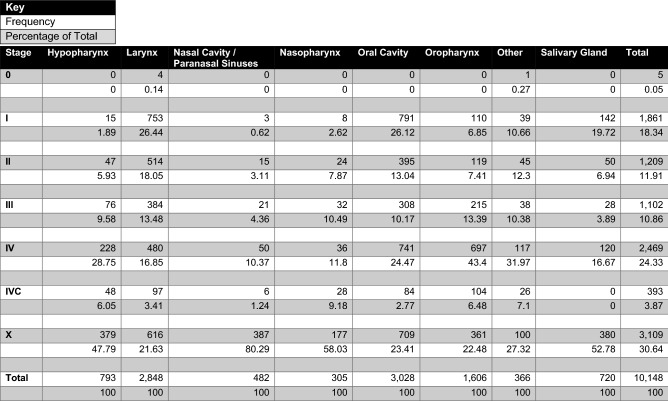
Overall and stratified by site statistics on disease stage at presentation

**Fig. 1:**
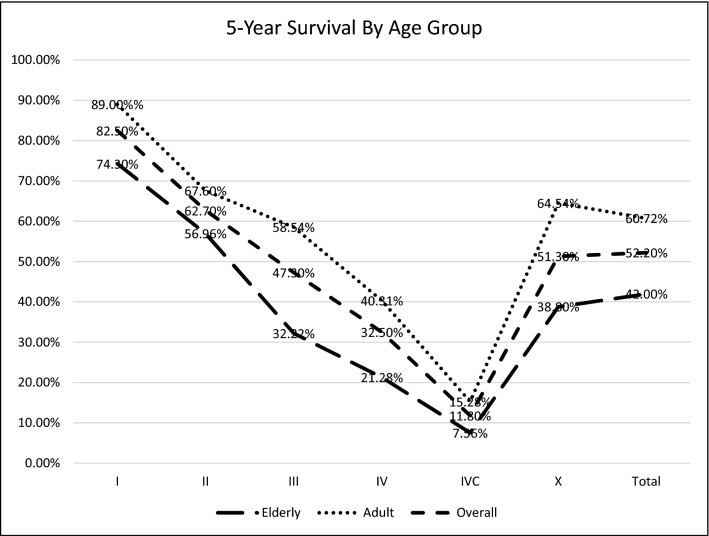
Head and neck cancer 5-year survival by stage and age group

5-year survival statistics by stage and age group (divided into the adult and elderly populations) are summarised in Fig. 1. The overall 5-year survival for HNC from 16 years of Irish data was 52.2%. 5-year survival from locoregionally confined disease (i.e. stage I and II) was 74.3%. The best prognosis was conferred by stage I disease, with 82.5% of patients surviving to 5 years. Stage IVC disease conferred the worst prognosis, with 46.5% alive at 1-year and 11.8% alive at 5 years.

A similar proportion of patients in both elderly and adult cohorts presented with each stage of disease. Survival was lower in the elderly cohort for all stages of disease. Formal record of disease stage was absent in 30.6% (*n* = 3109) of cases (represented as stage X). Following exclusion of cases which occurred in 2014, 55.2% (*n* = 1362) of cases where disease stage was not available occurred in elderly patients. The overall survival in this group, as shown in Fig. 1, is most analogous to stage III disease.

5-year survival by stage and year of incidence is presented in Table 5. Overall 5-year survival improved over the study period, with 46.5% of patients in 1994 surviving to 5 years compared to 56.8% in 2010. All stages of disease showed improvements in survivability including stage III disease, from 45.3% in 1994 to 58.2% in 2010, and stage IV disease, from 24.1% in 1994 to 38.4% in 2010.

**Table 5:** 5-year survival by stage and year of incidence

YOI	Stage I	Stage II	Stage III	Stage IV	Total
1994	69.6%	51.1%	45.3%	24.1%	46.5%
1995	67.3%	60.8%	47.6%	18.8%	46.7%
1996	80.4%	67.4%	41.9%	23.2%	48.2%
1997	85.0%	62.5%	44.4%	19.1%	49.3%
1998	72.4%	66.1%	39.0%	31.1%	52.3%
1999	72.9%	57.1%	37.5%	17.5%	45.2%
2000	89.1%	57.6%	31.0%	27.1%	48.6%
2001	92.5%	57.1%	42.4%	36.9%	52.6%
2002	82.9%	63.2%	57.1%	18.0%	49.7%
2003	80.7%	56.5%	51.5%	28.0%	50.4%
2004	86.9%	60.0%	44.8%	22.4%	50.1%
2005	88.6%	61.8%	44.7%	27.6%	56.3%
2006	84.0%	65.4%	50.0%	31.0%	54.2%
2007	88.5%	71.7%	44.2%	40.0%	57.8%
2008	82.4%	74.6%	57.1%	37.2%	60.4%
2009	84.8%	64.7%	52.1%	31.7%	54.0%
2010	85.7%	57.6%	58.2%	38.4%	56.8%

Survival statistics by site and by year of incidence are detailed in Table 6. Overall survival was highest in oral cavity cancer (58.9% alive at 5 years) closely followed by salivary gland and laryngeal cancer (58.6% and 58.6% alive at 5 years, respectively) and was worst with hypopharyngeal cancer (22.2% alive at 5 years). Oral cavity, salivary gland, and laryngeal malignancies all saw mild improvements in 5-year survival over the study period, while oropharyngeal and hypopharyngeal cancer survival improved significantly; 33.3% of oropharyngeal cancer patients survived to 5 years in 1994 compared to 55.1% in 2010 and 11.8% of hypopharyngeal cancer patients survived to 5 years compared to 36.7% in 2010.

**Table 6:** 5-year survival by site and year of incidence

YOI	Hypopharynx	Larynx	Nasal cavity/paranasal sinuses	Nasopharynx	Oral cavity	Oropharynx	Salivary gland	Total
1994	11.8%	55.3%	58.3%	42.9%	54.9%	33.3%	56.0%	46.5%
1995	17.9%	55.2%	50.0%	58.3%	52.3%	33.3%	62.5%	46.7%
1996	22.0%	59.3%	15.4%	30.8%	60.9%	34.2%	60.0%	48.2%
1997	15.2%	69.6%	52.6%	53.9%	55.2%	32.0%	46.7%	49.3%
1998	33.3%	57.1%	50.0%	50.0%	57.3%	41.7%	62.1%	52.3%
1999	23.1%	54.3%	53.9%	46.7%	53.3%	31.7%	38.1%	45.2%
2000	8.8%	61.1%	41.2%	70.0%	54.6%	34.0%	62.5%	48.6%
2001	27.3%	55.1%	53.9%	38.5%	63.5%	48.7%	40.0%	52.6%
2002	19.1%	52.5%	60.0%	50.0%	52.6%	43.1%	61.1%	49.7%
2003	32.3%	53.4%	70.6%	75.0%	56.1%	37.3%	48.2%	50.4%
2004	15.2%	60.8%	45.0%	69.2%	52.6%	43.8%	41.7%	50.1%
2005	21.2%	59.7%	50.0%	36.4%	65.3%	56.9%	67.9%	56.3%
2006	31.6%	52.9%	53.3%	38.5%	63.5%	52.6%	70.4%	54.2%
2007	14.3%	63.1%	61.1%	53.9%	67.2%	56.9%	70.0%	57.8%
2008	25.0%	63.8%	71.4%	50.0%	69.4%	48.4%	66.7%	60.4%
2009	27.1%	62.2%	46.7%	69.2%	56.5%	53.1%	58.1%	54.0%
2010	36.7%	58.7%	75.0%	56.5%	61.5%	55.1%	71.0%	56.8%
Total	22.2%	58.6%	54.3%	52.6%	58.9%	44.8%	58.6%	51.2%

## Discussion

### Histology

HNC is often considered analogous to SCC given the propensity for such lesions to develop in this region. This impacts significantly on management as a result of the relative radiosensitivity of SCCs as a group. From this cohort of 20 years of Irish patients, there was a lower proportion of SCC’s than would have been expected. This category represents 84.2% of patients, a figure that published epidemiological data would indicate should exceed 95% [3, 7, 17–19]. It should be noted that the NCRI database included patients of all ages, including paediatric patients in whom a higher proportion of malignancies such as sarcoma and rarer malignancies not coded for would be more prevalent than SCC. In addition, a further 8.1% of the cases represented in this database were coded as unspecified carcinoma. This group may well be composed of SCC variants such as spindle cell, verrucous, and basaloid carcinomas as well as rarer diagnoses such as lymphoma and olfactory neuroblastoma [20, 21].

### Gender

74.15% of patients in this cohort were male, reflecting the selectivity HNC displays for male gender worldwide. It has been reported that the risk of HNC is twofold to fivefold higher in men than in women, with factors such as ethnicity, socioeconomic deprivation, and alcohol and smoking prevalence predicting the risk balance in any given population. The overall ratio in the United States of America (USA) has previously been reported as around three to one, which is reflected by the Irish data [17, 22].

### Age

There was a broad age range represented in the reported data. While HNC is a rare diagnosis in the paediatric population, malignancy remains part of the differential for any mass in the head or neck. Previous data on paediatric HNC has indicated that where a single identifiable site can be identified, the most prevalent sites are the salivary glands, the nasopharynx, and the nasal cavity/middle ear [23, 24]. In this population, the latter of these findings were replicated, with patients under 20 presenting with nasopharyngeal, salivary gland, and nasal cavity/middle ear malignancies in 31%, 24%, and 20% of cases, respectively. Of equal note is the overall distribution of cases—48.15% of cases were in patients over the age of 65, the prespecified definition of an ‘elderly’ patient for this study. Clearly, a significant proportion of patients with HNC are chronologically elderly at the time of diagnosis. Such patients were notably less likely to have formal documentation of disease stage—whether this is coincidental or due to much reduced life expectancy and perceived futility of treatment in the frail patient remains unclear.

### Incidence

The incidence of HNC remained stable between 1994 and 2003. There was a steady increase in the number of HNC cases diagnosed each year in Ireland between 2004 and 2014, from 452 to 643. Worldwide, there has been a similar trend in HNC diagnoses, but the face of this entity is changing, with the incidence of malignancies at certain sites (such as laryngeal and nasopharyngeal) decreasing in frequency as a direct result of public health campaigns to reduce tobacco and alcohol consumption. There has been a concurrent rise in the incidence of oropharyngeal and hypopharyngeal malignancy due in no small part to the rising prevalence of HPV infection [25]. In the reported data there was no notable effect on the incidence of laryngeal or nasopharyngeal cancers before, during, or after the 2004 smoking ban came into effect in Ireland, though a small decrease in oral cavity cancer was noted. There has, however, been a gradual increase in the prevalence of oropharyngeal malignancies, from 13.59% in 1994 to 22.24% in 2014. HPV positive cancers are more common in white, socioeconomically privileged populations—this trend is unsurprising given the predominance of white ethnicity in Ireland and the relatively high socioeconomic status of the country as a whole [26].

The data presented in Table 3 also reflects the growing prevalence of HPV driven malignancies—the growing prevalence of young HNC patients has been well-described, and it has been shown that most HPC driven malignancies present at a younger age than their non HPV associated counterparts [27]. Equally worthy of note is the growing population of HNC patients over 85 years of age, a group whose numbers doubled over the 20 years of the study period.

### Stages and survival

The two chief components of the poor prognosis associated with HNC in general are the often advanced nature of the disease at presentation and the potential it possesses for locoregional or metastatic recurrence. It has previously been reported that up to 70% of HNC possesses some element of advanced disease at presentation [14]. 39.1% of patients presented with documented locoregionally advanced or metastatic disease in Ireland in the given period, but this number is likely an underestimation—30.6% of cases did not have documentation of their staging. While 643 of the 3109 cases represented here came about as a result of 2014 cases having no record of staging data, even when these are excluded, stage X disease still comprised 25.9% of the dataset. On further interrogation of this group, it contained a higher proportion of older patients than any other stage group. In addition, Table 4 demonstrates that patients with nasal cavity and paranasal sinus malignancies were overwhelmingly more likely than any other site not to have documented staging status, though salivary gland and nasopharyngeal malignancy were also overrepresented in this regard. It remains challenging to draw any definitive conclusions on this group, but it can be assumed that the predominant component of the group is advanced disease given the survival statistics in Fig. 1 place it somewhere closest to stage III in terms of prognosis. When patients who lacked formally documented staging were excluded, the proportion of patients who presented with locoregionally advanced or metastatic disease was 56.3%.

The overall 5-year cancer survival for HNC observed in the Irish population was 52.2%, though significant variability was observed when site and stage at presentation are considered. This is in line with HNC survival statistics for other European countries, and above the reported European average of 39.9%, though it should borne in mind that this figure includes data Eastern European where the survival was significantly lower than other regions [28, 29]. Stage I and II disease were associated with relatively high 5-year survival at 82.5% % and 62.7%, respectively. Stage IVC disease was associated with a dismal 5-year survival of 11.8%. Laryngeal cancer 5-year survival at 58.6% is on par with the European average of 58.9% while oral cavity 5 year-survival at 58.9% is well above the average of 45.4%, though this has been noted to be an area significantly contributed to by the overwhelming preponderance of such cases in Eastern Europe. Overall the best survival was associated with oral cavity, laryngeal, and salivary gland cancers, which is consistent with internationally reported survival statistics [8, 22, 30, 31].

Perhaps most noteworthy is the improvement in survival which has been observed in this time frame, most notable in the oropharynx and hypopharynx but also observed in the oral cavity, salivary glands, and larynx. This is at odds with much published international data, including a relatively recent paper from 2010 by Guntinas-Lichius et al. [32] who reported quite similar data on a large cohort of German HNC patients. Despite similar baseline characteristics, survival in this cohort was slightly lower overall and particularly in the oral cavity, larynx, and nasopharynx. Also described were similar data handling issues; specifically noted were a percentage of cases that lacked formally designated site data, though the prevalence of this was lower than the data presented in this study. More precise comparison of the differences in survival would be aided by more detailed staging data—as highlighted above the Irish data most likely underestimates the number of advanced cases owing to the number of stage X cases.

## Conclusion

There is a growing population of young HNC patients in Ireland driven largely by the growing prevalence of HPV associated malignancies. Survival is improving in Ireland but remains poor overall due to the often advanced nature of disease at presentation. The elderly HNC patient continues to pose challenges that are best resolved by close adherence to multidisciplinary assessment, development and encouragement of subspecialisation in cancer care, and use of targeted therapies based on tumour subsites.

## Declarations

### Availability of data and material

Stored and maintained by National Cancer Registry Ireland.

### Code availability

Stata 16.1.
